# Emergence of AI-Generated Multimedia: Visionary Physicists in Radiology Reincarnated

**DOI:** 10.7759/cureus.69471

**Published:** 2024-09-15

**Authors:** Ramin Javan, Navid Mostaghni

**Affiliations:** 1 Department of Radiology, George Washington University School of Medicine and Health Sciences, Washington, USA; 2 College of Medicine, California University of Science and Medicine, Colton, USA

**Keywords:** ai generated, ai-generated images, chatbots, dall-e, gpt-4, large language models, multimedia, radiology, video

## Abstract

AI-powered multimedia generation technologies, particularly video synthesis through stable diffusion and transformers, offer transformative potential for radiology education, communication, and visualization. This study explores various AI-generated multimedia categories, including image and video generation, as well as voice cloning, with a focus on video synthesis and future possibilities like scan-to-video generation. Utilizing tools such as *Midjourney*, *RunwaymL* *Gen2*, *D-ID*, and *ElevenLabs*, we aimed to reincarnate deceased influential physicists in radiology, demonstrating AI's capability to generate realistic content with accessible tools, fostering creativity and innovation in the radiology community.

We created 440 images through 110 prompts using image-to-image generation, 22 videos via image-to-video generation, and two videos showcasing text-to-voice and voice cloning techniques from December 1-7, 2023. Realism decreased from image-to-image to image-to-video and voiceover-to-video generations, with the latter requiring adjustments for lip, mouth, and head movements without incorporating facial expressions, eye movement, or hand motions.

Potential applications in radiology include improving and speeding up medical 3D visualization, as well as enhancing educational content, information delivery, patient interactions, and teleconsultations. The paper addresses limitations and ethical considerations associated with AI-generated content, emphasizing responsible use and interdisciplinary collaboration for further development.

These technologies are rapidly evolving, and future versions are expected to address current challenges. The ongoing advancements in AI-generated multimedia have the potential to revolutionize various aspects of radiology practice, education, and patient care, opening new avenues for research and clinical applications in the field.

## Introduction

“Wilhelm Conrad Roentgen discovered X-rays in 1895, revolutionizing medical imaging and leading to the development of radiography (Nobel Prize in 1901). Nikola Tesla's work on high-frequency currents laid the groundwork for various radiological devices, including MRI machines. He has been commemorated in the field with the International System (SI) unit of magnetic flux density named after him. Marie and Pierre Curie's research on radioactivity led to the development of radiation therapy for cancer treatment. At the same time, Marie was the first woman to win a Nobel Prize in 1903 in physics and was instrumental in breaking gender barriers in science and radiology. Albert Einstein was another founder of radiology because his theory of the photoelectric effect (Nobel Prize in 1921) explained how X-rays interact with matter. Christian Doppler's discovery of the Doppler effect is utilized in ultrasound imaging, allowing for the assessment of blood flow and other dynamic processes within the body.": GPT-4

Similar to large language models (LLMs), which use transformer-based technologies to generate human-like text, recent advancements in AI-powered multimedia generation have spawned a revolution in content creation. These are predominantly based on latent and stable diffusion modeling, which uses deep neural networks to gradually transform complex data through a series of probabilistic steps into desirable results, a process also known as iterative denoising [1,2].

The categories for AI-generated multimedia to consider are image generation, video synthesis, multi-view generation, voice generation, and 3D object generation. Image generation is made up of text-to-image and image-to-image generation. For video synthesis, which is the main focus of this manuscript, possible iterations include text-to-video, image-to-video, video-to-video, voiceover-video, and potentially, as it pertains to radiology, scan-to-video generation. Of note is that the term 'scan' as a speculative potential input source represents any cross-sectional radiologic exam, i.e., CT or MRI. On the more experimental end, multi-view generation has been of particular interest in AI development. With this technique, the AI can construct views of an object from multiple camera angles without entirely relying on raw data [3,4]. This technology poses an intriguing potential in 3D visualization, where a 3D rendering could be generated based on limited imaging data. This leads to 3D object generation, consisting of text-to-3D, image-to-3D, and possibly scan-to-3D utilizing AI-powered automated segmentation. Lastly, voice generation is made up of text-to-voice and voice cloning.

## Materials and methods

As mentioned earlier, the fundamental underlying algorithm behind the most commonly used tools is diffusion models. Other methods of tackling video synthesis have included variational RNNs, normalizing flows, autoregressive transformers, and generative adversarial networks (GANs) [5,6]. Transformers, particularly with autoregressive properties, are crucial in modeling sequential data, including videos, where each frame's generation depends on the previous ones [7]. GANs, in the broader context of video generation, provide a competitive framework for generating realistic images and videos by training two networks in tandem. However, results have generally been low-resolution, noisy, inconsistent, and short in duration [8,9]. With the increased availability of computational resources, higher-quality datasets that combine images and video have been used for advanced diffusion models. The stable diffusion algorithm, originally for image generation, is adapted to video generation by incorporating temporal dimensions, allowing for generating coherent video sequences from noise [10,11].

Stable video diffusion (SVD), developed by Stability.ai, is an example of a framework built upon latent diffusion models optimized for generating high-quality videos from textual or image inputs, introducing a robust motion representation beneficial for downstream tasks like multi-view generation. Multi-view generation denotes the ability to produce multiple consistent views of an object, thereby highlighting the model's understanding of 3D structures and perspectives. The overall process is accomplished using a three-phase methodology: text-to-image pretraining, which uses large image datasets to train the base model, ensuring it develops a strong foundational understanding of visual elements; video pretraining, which utilizes a curated set of videos at lower resolutions to introduce the model to temporal dynamics and motion representation; and finally, high-resolution video finetuning, which is the refinement of the model on a smaller, high-quality video dataset to enhance its capability to generate high-resolution videos [12].

The previously mentioned speculative concepts of AI-powered scan-to-video and scan-to-3D object generation, although not yet available, present an interesting future possibility in which a user can seamlessly create a video sequence from cross-sectional imaging data, i.e., CT and MRI, containing any desired anatomy and/or organ(s) by simply using textual instructions/prompts. This would allow non-radiologists, including surgeons, to independently create pre-operative planning 3D visualizations, alleviating radiologic technologists' workload.

A multitude of generative AI tools have recently become available with a wide range of capabilities. To showcase some of these capabilities in this manuscript, Midjourney v5.2 (image generator) [31], D-ID (voice-over video generator) [32], RunwayML Gen2 (video generator) [33], ElevenLabs (voice generator) [34], as well as GPT-4 (chatbot) [35] were implemented with the goal of reincarnating some of the most influential physicists whose work made significant contributions to the field of radiology. Beyond piquing interest and creativity amongst the radiology community, the reason for choosing this specific subject matter for content was two-fold. First, the diffusion algorithms are significantly more trained on general-purpose datasets such as photorealistic portraits than radiologic images or anatomically correct structures. Second, having the ability to create new content pertaining to these now-deceased individuals without the use of advanced graphic design and animation software that require advanced skills highlights the tremendous level of democratization that generative AI technologies have made possible beyond just LLMs. In this study, Midjourney was used instead of other image generators, such as Dall-E, due to its advanced photorealism capabilities as well as more advanced post-generation modification capabilities, at least at the time of testing (December 2023).

Stable diffusion algorithms have also been used to produce sounds and music based on a prompt but with limited early success. This was due to the requirement of segmenting audio training datasets due to the vast amount of information contained in sound files. Stabilty.ai's Stable Audio circumvented this issue by simplifying sounds and music so that the algorithm can be trained on hundreds of thousands of complete sound files, with their respective beginning, middle, and end. This new method generated more cohesive and desirable results, greatly resembling the original input [13]. 

## Results

Here, we demonstrate the text-to-image, image-to-image, image expansion, text-to-voice, voice cloning, and image-to-video capabilities of the aforementioned generative AI tools in order to bring to life visionary physicists that were deeply influential in the development and growth of radiology.

**Figure 1:**
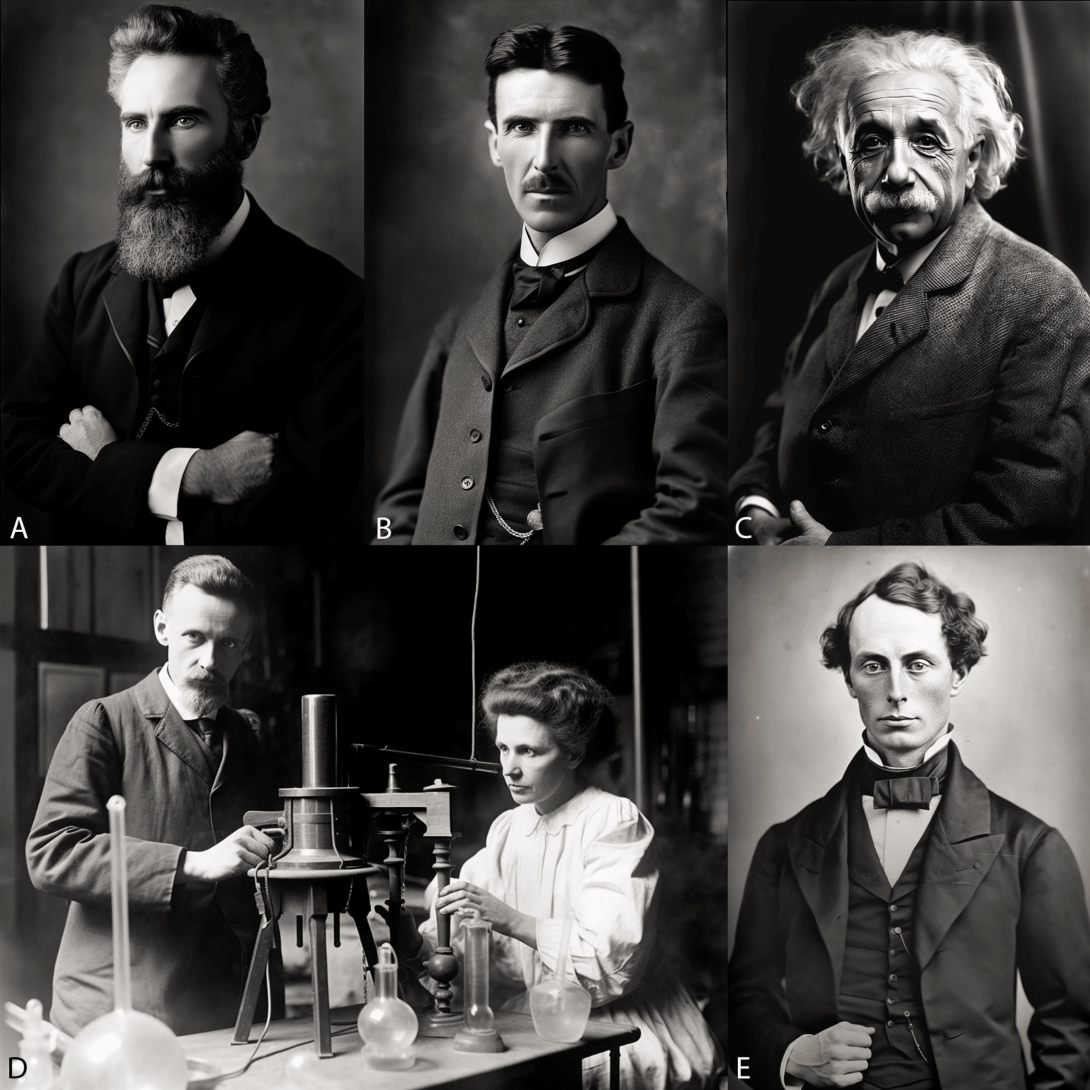
Visionary physicists in radiology AI-generated images using Midjourney v5.2 for generating photorealistic portraits of A. Wilhelm Conrad Roentgen, B. Nikola Tesla, C. Albert Einstein, D. Pierre and Marie Curie, and E. Christian Doppler (top left to bottom right). A combination of image-to-image and text-to-image capabilities were deployed using the ‘/imagine’ command, incorporating ‘--style raw’ and ‘--iw 2’ parameters. The ‘zoom out’ and ‘extend down’ capabilities were implemented to generate the lower half of the portraits showing the upper body. Of note, for Christian Doppler’s image, the ‘/blend’ command was implemented by blending a photograph and a sketch to generate more suitable results.

**Figure 2:**
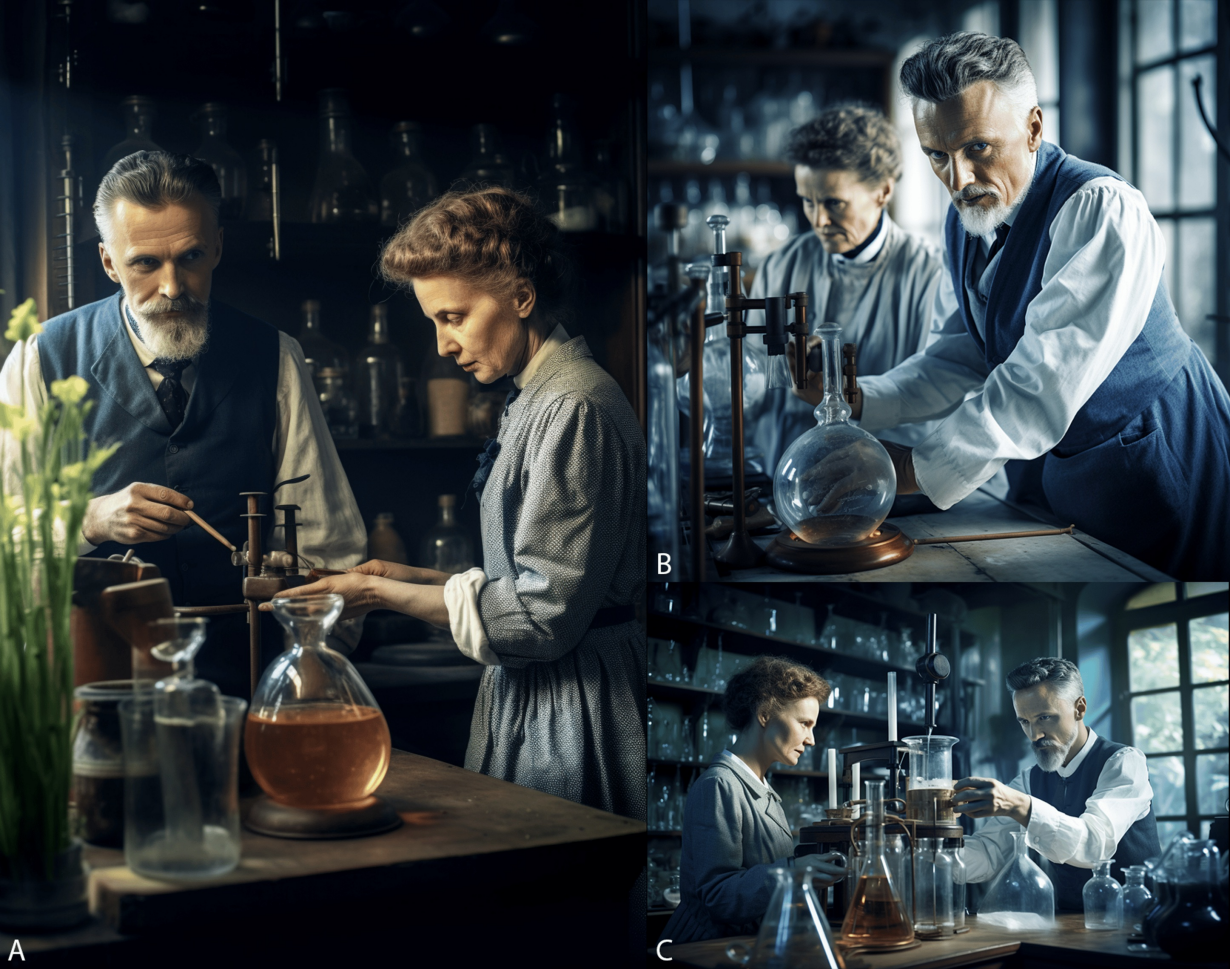
Marie and Pierre Curie working in the laboratory A-C. Demonstration of AI’s capability in generating photorealistic images using a combination of image-to-image and text-to-image prompts. The prompt used was: “https://s.mj.run/88YJfgxIj-o color photograph of Marie Curie and Pierre Curie working in their lab in realistic colors, Sony a7rIII, lens 35mm f4, photorealism, detailed, 8k --style raw --iw 0.3 --ar 2:3”. Parameter ‘--style raw’ refers to the photorealism type of image, ‘--iw' refers to the weight the AI should take into consideration for the input image in its generation vs. the text prompt, with 2 being the maximum weight towards the input image and 0 being significantly skewed towards the input text, and ‘--ar' refers to the aspect ratio. Note the blurred face of Marie Curie vs. the sharp in-focus face of Pierre Curie in the top right image, a characteristic of a photograph taken with a f-stop of 4 as instructed.

**Figure 3:**
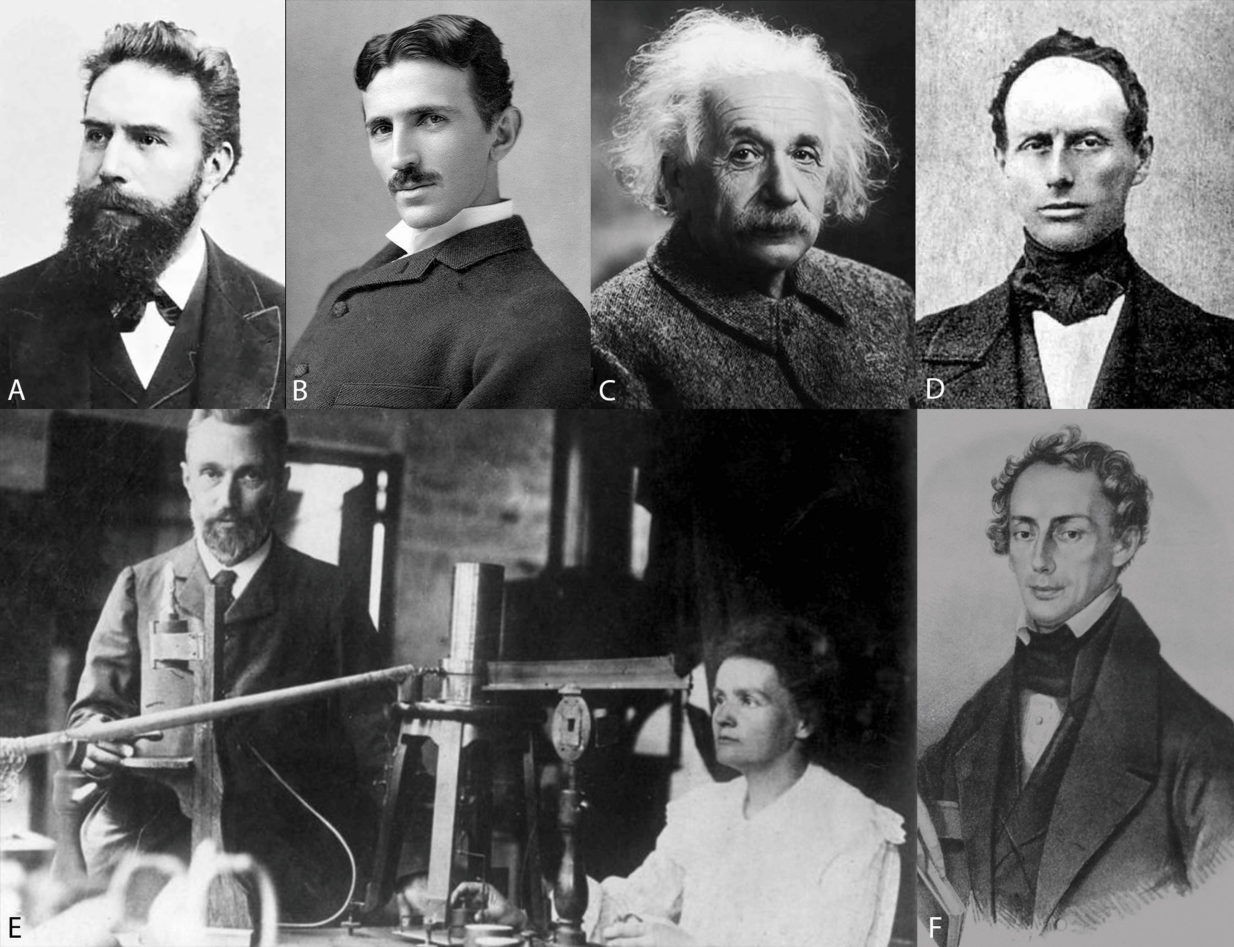
Original images from Wikipedia A. Wlihelm Conrad Roentgen photograph, B. Nikola Tesla photograph, C. Albert Einstein photograph, D. Christian Doppler photograph, E. Pierre and Marie Currie photograph, F. Chrisitan Doppler vintage sketch. (All images are in the public domain, obtained from Wikipedia)

A combination of image-to-image and text-to-image capabilities was deployed in Midjourney v5.2 using the ‘/imagine’ command (Figures 1A-1E, 2A-2C). Original photos of influential physicists in radiology were obtained from Wikipedia (Figures 3A-3F) and provided to mid-journey for image-to-image generation. The ‘zoom out’ and ‘extend down’ capabilities were implemented to generate the lower half of the portraits to eventually show the rest of the upper body de novo based on the style of the subject matter’s historical time period. Of note, for one of the images, the ‘/blend’ command was implemented by combining features of a photograph and a sketch to be able to generate a more suitable result (Figures 3D, 3F), since the original vintage photograph was low-quality and initial results did not resemble the original photograph enough, overly accentuating features such as the forehead height and jaw width. This highlights an important aspect of using generative AI, which is the need for constant trial and error through prompting techniques while utilizing the multitude of settings, features, and custom capabilities that are available in a given tool. A total of 440 images were generated from 110 prompt/iteration combinations (December 1-7, 2023) in order to arrive at desirable results.

AI-generated video with generic voiceover (Video 1) was created using D-ID from an initial image generated using Midjourney v5.2, showcasing the ability to animate a still portrait to match and simulate the enunciation of words within the accompanying desired text. This tool is limited in that it only adjusts the movements of the mouth, lips, and slightly the head without the incorporation of facial expressions, head movement, and hand motion, therefore significantly reducing its realism. However, this is a new technology and is currently undergoing rapid development, and future iterations should gradually address these issues. Alteration of a native video to mimic the enunciation of any desired text has also been made possible in addition to completely replacing the face of the subject, a phenomenon known as deepfake [14]. 

**Video 1:** Wilhelm Conrad Roentgen AI-generated video using D-ID from an initial image generated using Midjourney v5.2. “If the hand be held between the discharge-tube and the screen, the darker shadow of the bones is seen within the slightly dark shadow-image of the hand itself... For brevity's sake I shall use the expression 'rays'; and to distinguish them from others of this name I shall call them 'X-rays'.”

Animating an image of Pierre and Marie Curie to mimic a short video clip, i.e., image-to-video generation, was achieved using RunwayML Gen2 (Video 2, 3). For the initial input color image, image-to-image generation was performed using Midjourney v5.2 from a vintage black and white photograph (Fig 3E). To maintain maximum fidelity and similarity of the generated video to the input image, the accompanying text prompt in RunwayML Gen2 was left blank. Text-to-video prompts did not yield fully satisfactory results. After a total of 22 attempts (December 1-7, 2023), satisfactory results were obtained where the incorporated arbitrary motion led to the least noticeable artifacts, especially in the components of the face and hands, as well as lighting. 

**Video 2:** Pierre and Marie Curie in the laboratory in color (10:16 aspect ratio) Demonstration of image-to-image (Midjourney v5.2) and image-to-video (RunwayML Gen2) capabilities of generative AI.

**Video 3:** Pierre and Marie Curie in the laboratory in black & white (16:10 aspect ratio) Demonstration of image-to-image (Midjourney v5.2) and image-to-video (RunwayML Gen2) capabilities of generative AI.

Voice cloning, which entails the creation of spoken language based on any desired text to closely match a subject's voice, tone, accent, and pronunciation, was achieved by using ElevenLabs. The initial training input data consisted of real voice recordings of Albert Einstein from archived radio interviews. Due to the limited number of recordings and their low quality, which sometimes included background noise, extra steps were required to produce the cleanest sample, using Audacity [15] and CleanVoice.ai [16]. The textual content of the output voice was generated through GPT-4 by prompting it to speculate what Einstein would have opined about quantum computing and AI had he has been alive today, based on his extensive past writings and interviews, both in terms of style and thought process. Subsequently, D-ID was implemented for image-to-video generation using a real photograph of a younger version of Einstein (Video 4). The photos of the most well-known appearance of an aged Einstein could not be used due to the restrictions and alignment rules incorporated in D-ID, preventing users from creating videos of prominent or public figures [17]. 

**Video 4:** A young Albert Einstein discussing quantum computing and AI Demonstration of voice cloning (ElevenLabs) from the real voice of Einstein and image-to-video (D-ID) generation from a real photograph of Einstein (Wikipedia). "Quantum computing, a perplexing extension of the very quantum mechanics I once questioned, opens doors to unimaginable calculations. Artificial Intelligence, in its pursuit of mimicking human thought, shows both the brilliance and audacity of our scientific endeavors. Yet, we must tread with caution, for in our quest to know and control everything, we risk losing sight of our humanity and ethical compass." GPT-4’s speculation of what Einstein would say about quantum computing and AI if he were alive today. A young version of Einstein was mimicked as D-ID would not accept a photo of Einstein at an older age that he is best known by for safety and ethical reasons.

## Discussion

Technological advancements in the medical industry have progressed quickly in recent years and are consistently changing. One of the most groundbreaking advancements came with the implementation of the 'Internet of Things' concept in the medical field [18]. As these nascent technologies continue to develop rapidly, future iterations will continuously improve. OpenAI's newest video generation model, Sora, released in February 2024, can produce unprecedented minute-long, convincingly realistic, and high-quality samples. This is particularly an important achievement since video generative models, much like audio models, struggle with maintaining consistency and comprehensiveness of prolonged samples [19]. In the field of radiology, such generative AI technologies potentially present opportunities to enhance education, 3D visualization, patient communication, and research. Of note, the iterative process of "stable diffusion" is used in order to generate a final image from an initial image of "random noise". An example of the progression through this process is provided in Video 5.

**Video 5:** Generation of Nikola Tesla through stable diffusion This clip shows the underlying mechanism used in Midjourney for image generation known as stable diffusion, an iterative denoising process from an initial seed of random noise.

Potential applications in radiology

Smoothing frame transitions in video: AI can generate intervening frames in a video sequence, reducing the need for extensive raw data acquisition. For 3D rotation videos, for example, AI can interpolate frames [20] to create smoother motion, reducing computational resource requirements. AI can potentially increase frame rates in video sequences, such as fluoroscopy or ultrasound, making the visualization of movement more fluid and easier to analyze while reducing radiation exposure and scanning time.

Improving medical 3D visualization: Incorporation of AI-powered hyper-realism [21], stylization, noise reduction, sharpening, color saturation, and improvements in lighting, contrast, and resolution may be possible, further enhancing cinematic rendering capabilities. This could enable robust, high-resolution interactive 3D visualization while reducing the need for raw data processing and segmentation during 3D reconstruction.

Virtual reality (VR) and augmented reality (AR): AI can enable masking, tagging, and labeling of anatomic structures in videos. Immersive VR environments for preoperative planning may be created, allowing radiologists and surgeons to explore 3D reconstructions of patient anatomy and simulate interventions. AI-generated AR overlays on live patient images during procedures can provide real-time guidance [22], anatomical references, and critical information to improve outcomes and training.

Enhancing trainee education: Customized educational content, such as interactive tutorials and simulations, may be created faster and easier and tailored to different learning styles and levels [23]. Generating realistic patient interactions and contrast reaction scenarios is another possibility, helping trainees' and professionals' management and communication skills in a risk-free environment.

Personalized patient interaction: Generating personalized multimedia [24] content for patients, explaining their conditions, treatment options, and what to expect from procedures using accessible data, visuals, and desired language is an intriguing possibility. AI-generated enhanced images or videos that highlight pathological findings or simulate the effects of disease progression or potential treatments can be an aid in patient consultations.

Teleconsultation enhancements: Video streaming adjustments such as digitally assisting in maintaining eye contact [25], eliminating background noise and distractions, masking sensitive data, and providing live transcription/translation are possible. Integrating on-demand AI-generated images and animations during teleconsultations may allow for better explanations of radiological findings and intervention plans while consulting with other specialists, such as during tumor boards.

Video summarization: For lengthy videos of conferences, meetings, rounds, tumor boards, and lectures, AI can summarize [26] key findings and moments into a shorter video, streamlining review processes for trainees or radiologists, therefore increasing efficiency.

Data visualization: Complex radiomics and scientific datasets can be transformed into interactive, tagged, graphical visualizations, making it easier to identify patterns, correlations, and anomalies in research and during quality control.

Piquing interest in radiology: AI-generated interactive content for medical students can make radiology more engaging, encouraging more students to consider it a career path early on [27]. It can empower radiologists to create content that can be used for social media campaigns, health policy, and advocacy endeavors, as well as raising awareness in the general public to promote preventive imaging such as screening mammograms. It could allow radiologists and technicians to help young pediatric patients undergo imaging exams more easily by generating on-demand content tailored to a child's specific interests [28].

Effective information delivery: AI can automate the creation of on-demand informative multimedia for patients and professionals using an individual radiologist's chosen library of up-to-date documents, even mimicking one's voice and mannerisms. This type of content deliverable on mobile phones, be it a combination of images with audio and/or video, may present a more digestible medium [29]. Compared to a purely written format, this could make recommendations more likely to be followed, which in turn could lead to improved compliance and follow-up care [30].

Reliability of content, consistency across multiple generated pieces, and bias propagation are some of the major limitations to consider. Video quality, presence of artifacts, and length limitations are other pitfalls that are being addressed with newer iterations. Perhaps the biggest concern with AI-generated media is the potential for misinformation, fraud, and misinterpretation, highlighting the immense amount of effort required by the industry and governments to ensure safe and responsible use. Issues of copyright and consent lead to legal challenges and ethical dilemmas. Lastly, substantial computational resources are inherently required for these tools, potentially limiting accessibility for individuals or organizations with fewer resources.

## Conclusions

AI-powered multimedia generation technologies present a transformative opportunity for the field of radiology. Our examination of image generation, video synthesis, and voice cloning demonstrates the potential of these tools to revolutionize education, communication, and visualization in radiology. While the current iterations show promising results, they also highlight areas for improvement. As these technologies rapidly evolve, we anticipate significant advancements that will address current limitations and unlock new possibilities. However, it is crucial to approach these developments carefully, considering ethical implications and responsible use through continued interdisciplinary collaboration among radiologists, AI researchers, and ethicists, ultimately enhancing medical education and improving patient care.
